# Oil palm phenolics confer neuroprotective effects involving cognitive and motor functions in mice

**DOI:** 10.1179/1476830512Y.0000000047

**Published:** 2013-09

**Authors:** Soon-Sen Leow, Shamala Devi Sekaran, YewAi Tan, Kalyana Sundram, Ravigadevi Sambanthamurthi

**Affiliations:** 1 Malaysian Palm Oil Board, No. 6, Persiaran Institusi, 43000 Kajang, Selangor, Malaysia; 2 University of Malaya, Kuala Lumpur, Malaysia; 3 Malaysian Palm Oil Council, 47301 Kelana Jaya, Selangor, Malaysia

**Keywords:** Antioxidants, Gene expression, Microarray, Neurodegeneration, Oil palm phenolics

## Abstract

**Objectives:**

Phenolics are important phytochemicals which have positive effects on chronic diseases, including neurodegenerative ailments. The oil palm (*Elaeis guineensis*) is a rich source of water-soluble phenolics. This study was carried out to discover the effects of administering oil palm phenolics (OPP) to mice, with the aim of identifying whether these compounds possess significant neuroprotective properties.

**Methods:**

OPP was given to BALB/c mice on a normal diet as fluids for 6 weeks while the controls were given distilled water. These animals were tested in a water maze and on a rotarod weekly to assess the effects of OPP on cognitive and motor functions, respectively. Using Illumina microarrays, we further explored the brain gene expression changes caused by OPP in order to determine the molecular mechanisms involved. Real-time quantitative reverse transcription-polymerase chain reaction experiments were then carried out to validate the microarray data.

**Results:**

We found that mice given OPP showed better cognitive function and spatial learning when tested in a water maze, and their performance also improved when tested on a rotarod, possibly due to better motor function and balance. Microarray gene expression analysis showed that these compounds up-regulated genes involved in brain development and activity, such as those under the regulation of the brain-derived neurotrophic factor. OPP also down-regulated genes involved in inflammation.

**Discussion:**

These results suggest that the improvement of mouse cognitive and motor functions by OPP is caused by the neuroprotective and anti-inflammatory effects of the extract.

## Introduction

Harmful free radicals and reactive oxygen species play an important role in aging and chronic diseases.^1^ Thus, many preventive measures which involve efforts to offer resistance against oxidative stress, such as physical activity and dietary intervention, have been suggested to combat the advancement of chronic diseases as we age. Oxidative damage is particularly detrimental to the brain, where the neuronal cells are largely post-mitotic and those which are damaged cannot be readily replaced via mitosis.^2^ During normal aging, the brain undergoes morphological and functional changes resulting in the observed neurobehavioral declines such as decrements in cognitive and motor performance, which leads to Alzheimer's disease and Parkinson's disease respectively.

Diets containing high amounts of phytochemicals can provide protection against these free radical-induced diseases, due to their high antioxidant activities. Plant phenolics are important antioxidants because of their high redox potential, which allows them to act as reducing agents, hydrogen donors, singlet oxygen quenchers, and metal chelators. Antioxidants that accumulate in neuronal tissues are potential candidates for the prevention and treatment of neuronal disorders involving oxidative stress. Phenolic antioxidants may or may not cross the brain barrier, depending on their properties, such as charged state, lipophilicity, and interactions with efflux transporters, with possible relative specificity of the compounds for different brain areas.^3^ For example, proanthocyanidins from blueberries may be found in the striatum while ellagitannins from strawberries may involve the hippocampus.^4^ Many recent studies have shown that plant phenolics have neuroprotective effects, through their ability to reduce oxidative stress.^2,4–11^

In addition to their antioxidant activities, plant phenolic compounds such as flavonoids have been shown to possess other molecular mechanisms,^12^ some of which are neuroprotective. One of these is the modulation of brain cell signaling pathways, such as the activation of extracellular signal-regulated protein kinase (ERK) and the protein kinase B (PKB/Akt) signaling pathways, leading to the activation of the cyclic adenosine monophosphate-response element-binding protein (CREB), a transcription factor responsible for increasing the expression of neurotrophins important in defining memory.^13^ A blueberry-containing diet, for example, has been shown to improve the performance of aged animals in spatial working memory tasks via the activation of CREB and increases in brain-derived neurotrophic factor (BDNF) levels in the hippocampus.^14^ In addition to affecting signaling pathways, the effects of plant phenolics on learning and memory may also be mediated by peripheral and cerebral vascular effects which lead to the growth of new neurons.^13^

On the basis that oil palm phenolics (OPP)^15^ possess significant antioxidant activities and various biological effects,^16–20^ we hypothesized that they may also have significant neuroprotective effects *in vivo*. Thus, we fed BALB/c mice a normal diet with OPP supplemented as fluids for 6 weeks and looked for signs of improvement in brain functions. This included testing the mice in a water maze to identify whether there was improvement in cognitive function and spatial learning, as well as testing them on a rotarod in order to identify effects on motor function and balance. We then carried out microarray analysis on the brains of these mice in order to determine the molecular mechanisms involved in conferring the neuroprotective properties of OPP. The microarray data were further validated with real-time quantitative reverse transcription-polymerase chain reaction (qRT-PCR) experiments.

## Methods

### OPP samples

OPP samples were prepared according to the methods described in Sambanthamurthi *et al.*^15^ OPP contains numerous phenolic acids. Three isomers of caffeoylshikimic acid are major components of the extract. Other phenolic acids include caffeic acid, protocatechuic acid, and *p*-hydroxybenzoic acid. The detailed composition of OPP is as described earlier.^16^

### Mouse feeding and organ harvesting

All male inbred BALB/c mice which were designated for this study were purchased from the Institute of Medical Research, Kuala Lumpur, Malaysia, at around 5 weeks of age just after weaning. All animal procedures were approved by the Animal Care and Use Committee of the University of Malaya, Kuala Lumpur, Malaysia. The animals were randomly assigned into cages (five animals per cage) and acclimatized for 1 week, during which a standard chow diet purchased from the University of Malaya, and distilled water were given *ad libitum*.

At the start of the experiment, the diet of the animals was changed to a custom-made normal diet (58.2% kJ/kJ carbohydrate, 27.2% kJ/kJ protein, and 14.6% kJ/kJ fat, including cellulose, mineral mix, vitamin mix, and dl-methionine). The control group (*n = 5*) was given distilled water while the treatment group (*n = 5*) was supplemented with OPP in the drinking fluids. The antioxidant content of the OPP given was around 1500 ppm gallic acid equivalent. Food and fluids were changed daily.

As shown in previous studies, the volume of OPP taken by an approximately 25 g mouse was around 1.75 ml/day.^19,20^ The amount of OPP consumed would thus be around 2.625 mg (in gallic acid equivalent weight). This would be equivalent to the consumption of about 350 ml (in volume) or 500 mg (in gallic acid equivalent weight) of OPP by a 60 kg human, calculated based on the body surface area normalization method.^21^ Previous studies have also shown that no significant differences were observed in terms of the food and fluid intakes of mice from week to week.^19,20^

Mice were subjected to water maze and rotarod trials once a week throughout the feeding period. After 6 weeks of *ad libitum* feeding, the mice were sacrificed via euthanasia with diethyl ether followed by exsanguination. Their brains were excised, blotted, snap-frozen in liquid nitrogen, and stored at −80°C until the total RNA extraction process for gene expression analysis.

### Water maze: cognitive function and spatial learning

Mice were tested in a water maze every week throughout the feeding period. These experiments were carried out in a room with no windows and only one door. The maze painted black (100 cm diameter and 60 cm height) was filled with water 1 cm above a transparent platform (11 cm diameter and 30 cm height), at a temperature of 26°C. The platform was placed at a permanent position in the middle of the north-east quadrant of the maze. During the trials, each mouse was released with its head pointed towards the side of the water maze at the same four predetermined starting positions near the south-west quadrant of the maze (south-east, south, west, and north-west), so that all four positions were used.^22^ Various external cues were available and visible to each mouse swimming in the water maze, such as the door, lights, several experimental equipment which was seldom used (centrifuges and vacuum concentrators permanently positioned on bench tops), the computer, and the experimenter. The experimenter who handled the mice and carried out the experiments was always the same person and was a non-smoker. When the experiment was carried out, the door was closed at all times and the experimenter always remained at a constant location near the south-west quadrant of the maze during the trials. Therefore, no sources of interference were present. Each mouse was tracked by using a Panasonic WV-CP280/G color closed-circuit television camera (Panasonic Corporation, Osaka, Japan) suspended 90° about 200 cm above the center of the water level and connected to an Euresys Picolo Diligent sn/219 frame grabber board (Euresys s.a., Liège, Belgium) installed in a desktop with Microsoft^®^ Windows XP (Microsoft Corporation, Redmond, WA, USA).

Each trial started 3 seconds after a mouse was in the arena (which was defined as the circular water border) and stopped at a maximum duration of 60 seconds (after which the mouse was guided to the platform and left to stay there for 10 seconds), or 10 seconds after the mouse found and stayed on the platform. The latency to the platform, the mean distance to the platform, and the mean velocity of each mouse was recorded by the Ethovision XT video tracking system and software (Noldus Information Technology, Wageningen, The Netherlands). Each mouse was tested four times during each time point with a rest period of about 5 minutes in between trials. Each mouse was towel dried after each trial before it was returned to a holding cage. The first trial was excluded from analysis as it served the purpose of acclimatizing the mouse to the water maze after a week's gap from the last trial. The remaining three trials for each mouse were averaged and statistical analysis using a two-tailed unpaired Student's *t*-test was carried out on these average values in the Microsoft Excel software (Microsoft Corporation), with *P* values of less than 0.05 considered statistically significant.

### Rotarod: motor function and balance

Mice were tested on a five-laned IITC 755 Series 8 rotarod (IITC Life Science, Inc., Woodland Hills, CA, USA) every week throughout the feeding period. The rotarod was started at 4 rpm and accelerated to 40 rpm over a period of 5 minutes in forward mode, with a rest period of about 3 minutes in between trials. Each mouse was tested four times during each time point, but the first trial was excluded from analysis as it served the purpose of acclimatizing the mouse to the rotarod after a week's gap from the last trial. The remaining three trials for each mouse were averaged and statistical analysis using a two-tailed unpaired Student's *t*-test was carried out on these average values in the Microsoft Excel software (Microsoft Corporation), with *P* values of less than 0.05 considered statistically significant.

### Microarray studies: gene expression analysis

Total RNA isolation from mouse brains was first carried out using the RNeasy Mini Kit (Qiagen, Inc., Valencia, CA, USA) and QIAshredder homogenizer (Qiagen, Inc.), according to the manufacturer's instructions. The total RNA samples obtained were subjected to NanoDrop 1000A Spectrophotometer (Thermo Fisher Scientific, Waltham, MA, USA) measurement for yield and purity assessment. Integrity of the total RNA samples was then assessed using the Agilent 2100 Bioanalyzer (Agilent Technologies, Santa Clara, CA, USA) and Agilent RNA 6000 Nano Chip Assay Kit (Agilent Technologies). Four total RNA samples with the highest RNA Integrity Numbers and 28S/18S rRNA ratios within each condition (either control or treatment) were then selected for microarray studies.

Amplification of total RNA samples which were of high yield, purity, and integrity was then carried out using the Illumina TotalPrep RNA Amplification Kit (Ambion, Inc., Austin, TX, USA) according to the manufacturer's instructions. The cRNA produced was then hybridized to the Illumina MouseRef-8 Version 1 Expression BeadChip (Illumina, Inc., San Diego, CA, USA), using the Direct Hybridization Kit (Illumina, Inc.). Microarray hybridization, washing, and scanning were carried out according to the manufacturer's instructions. The raw gene expression data obtained are available at Gene Expression Omnibus^23^ (accession number: GSE40030).

Quality control of the hybridization, microarray data extraction, and initial analysis were carried out using the Illumina BeadStudio software (Illumina, Inc.). Outlier samples were removed via hierarchical clustering analysis provided by the Illumina BeadStudio software (Illumina, Inc.) and also using the TIGR MeV software (Institute for Genomic Research, Rockville, MD, USA),^24^ via different distance metrics. Three replicates per condition (with outliers removed) were then considered for further analysis. Gene expression values were normalized using the rank invariant method and genes which had a detection level of more than 0.99 in either the control or treatment samples were considered significantly detected. To filter the data for genes which changed significantly in terms of statistics, the Illumina Custom error model was used and genes were considered significantly changed at a |differential score| of more than 13, which was equivalent to a *P* value of less than 0.05.^25^

The genes and their corresponding data were then exported into the Microsoft Excel software (Microsoft Corporation) for further analysis. To calculate fold changes, an arbitrary value of 10 was given to expression values which were less than 10. Fold changes were then calculated by dividing means of Signal Y (treatment) with means of Signal X (control) if the genes were up-regulated and *vice versa* if the genes were down-regulated. Two-way (gene and sample) hierarchical clustering of the significant genes was then performed using the TIGR MeV software (Institute for Genomic Research)^24^ to ensure that the replicates of each condition were clustered to each other. The Euclidean distance metric and average linkage method were used to carry out the hierarchical clustering analysis.

Changes in biological pathways and gene ontologies were assessed via functional analysis, using the GenMAPP^26^ and MAPPFinder^27^ softwares (University of California at San Francisco, San Francisco, CA, USA). The MAPPFinder software ranks GenMAPPs (pathways) and gene ontologies based on the hypergeometric distribution. Readers are referred to Doniger *et al.*^27^ for further explanations of the terms used in the MAPPFinder software. GenMAPPs and gene ontologies which had permuted *P* values of less than 0.05, numbers of genes changed of more than or equal to 2, and *Z* scores of more than 2 were considered significant. Up-regulated and down-regulated genes were analyzed separately in this functional enrichment analysis. It should be noted that the MAPPFinder software clusters multiple probes for a distinct gene into a single gene grouping in order to calculate the number of distinct genes which meet the user-defined criteria, not the probes.

Changes in regulatory networks were also analyzed through the use of the Ingenuity Pathways Analysis software (Ingenuity^®^ Systems, Redwood City, CA, USA). A dataset containing differentially expressed genes and their corresponding fold changes was uploaded into the application. Analyses of up-regulated and down-regulated genes were carried out separately. Each gene identifier was mapped to its corresponding gene object in the Ingenuity Pathways Knowledge Base. These genes were overlaid onto a global molecular network developed from information contained in the Ingenuity Pathways Knowledge Base. Networks of these focus genes were then algorithmically generated based on their connectivity. A network is a graphical representation of the molecular relationships between genes or gene products. Genes or gene products are represented as nodes, and the biological relationship between two nodes is represented as an edge (line). The intensity of the node color indicates the degree of up-regulated or down-regulation. Nodes are displayed using various shapes that represent the functional class of the gene product. Edges are displayed with various labels that describe the nature of the relationship between the nodes.

### Real-time qRT-PCR studies: validation of microarray data

Two-step real-time qRT-PCR studies were carried out on two target genes, *Fos* (Finkel–Biskis–Jinkins osteosarcoma) and *Bcar1* (breast cancer anti-estrogen resistance 1), using TaqMan Gene Expression Assays (Applied Biosystems, Foster City, CA, USA). The same aliquots of total RNA samples used in the microarray experiments were utilized for this analysis. Primer and probe sets for the selected genes were obtained from the Applied Biosystems Inventoried Assays-On-Demand (Applied Biosystems).

Briefly, reverse transcription to generate first-strand cDNA from total RNA was carried out using the High-Capacity cDNA Reverse Transcription Kit (Applied Biosystems). Real-time PCR was then carried out on the first-strand cDNA generated using a 25 µl reaction volume in an Applied Biosystems 7000 Real-Time PCR System (Applied Biosystems) using the following conditions: 50°C, 2 minutes, 1 cycle; 95°C, 10 minutes, 1 cycle; 95°C, 15 seconds, and 60°C, 1 minute, 40 cycles. Reactions for each biological replicate and non-template control (NTC) were carried out in duplicates.

Quality control of the replicates used, real-time qRT-PCR data extraction, and initial analysis were carried out using the 7000 Sequence Detection System software (Applied Biosystems). A manual threshold of 0.6000 and an auto baseline were applied in order to obtain the threshold cycle (Ct) for each measurement taken. The threshold was chosen as it intersected the exponential phase of the amplification plots.^28^ The criteria for quality control of the data obtained include ΔCt of less than 0.5 between technical replicates and ΔCt of more than 5.0 between samples and NTCs.^29^

Relative quantification of the target genes of interest was carried out using the qBase 1.3.5 software (Center for Medical Genetics, Ghent University Hospital, Ghent, Belgium),^30^ which takes into account the calculations of amplification efficiencies and multiple housekeeping genes. Expression levels of target genes were normalized to the geometric mean of three housekeeping genes, *Sfrs9*, *Guk1*, and *Hnrpab*. The stability of these housekeeping genes was assessed using the geNorm 3.5 software (Center for Medical Genetics, Ghent University Hospital).^31^ Statistical analysis of the data obtained was carried out using a two-tailed unpaired Student's *t*-test in the Microsoft Excel software (Microsoft Corporation), with *P* values of less than 0.05 considered statistically significant.

## Results

**Figure 1:**
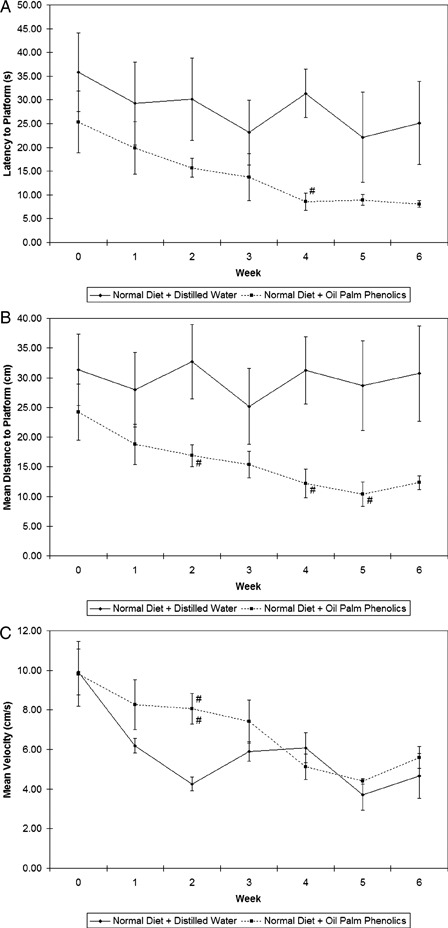
Results of water maze trials. (A) Latency to platform; (B) mean distance to platform; (C) mean velocity. ^#^*P* < 0.05 versus normal diet + distilled water; *n* = 5; mean ± SEM.

The water maze is based upon the premise that animals have evolved an optimal strategy to explore their environment and escape from water with minimum effort by swimming the shortest distance possible. Fig. 1 shows the results of the water maze trials, in which mice on the normal diet given OPP showed a downward trend in latency to the platform (Fig. 1A) and mean distance to the platform (Fig. 1B) from week to week while mice in the control group showed somewhat constant values throughout the weeks. Although the mean velocity values of the mice in the treatment group were slightly increased at the beginning of the trials compared to those of the controls (Fig. 1C), the differences were not significant anymore towards the end of the study, thus indicating an improvement in spatial learning instead of swimming speed in these mice.

**Figure 2:**
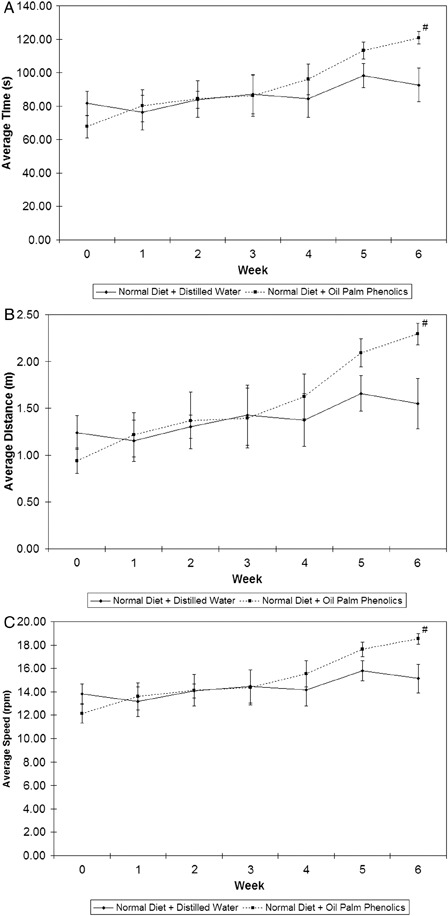
Results of rotarod trials. (A) Average time; (B) average distance; (C) average stopping speed. ^#^*P* < 0.05 versus normal diet + distilled water; *n* = 5; mean ± SEM.

The function of the rotarod is to test the balancing abilities of mice. As shown in Fig. 2, mice on the normal diet supplemented with OPP had improved balance and motor coordination, as they demonstrated a more obvious upward trend in terms of average time (Fig. 2A), average distance travelled (Fig. 2B), and average stopping speed (Fig. 2C) before they fell off the rotating drums compared to the controls. Based on the trends therefore, we deduced that mice given OPP performed better in both the water maze test and the rotarod task.

In order to identify the possible molecular mechanisms involved in the neuroprotective properties of OPP, we carried out microarray analysis on brains harvested from mice in the control and treatment groups. The filtering criteria used resulted in 113 genes significantly up-regulated and 145 genes significantly down-regulated in brains of mice on a normal diet given OPP compared to the controls. The full list of significantly changed genes is given in Supplementary Material 1. GenMAPPs and gene ontologies considered significantly up-regulated are shown in Table 1, while those significantly down-regulated are shown in Table 2.

**Table 1:** GenMAPPs and gene ontologies significantly up-regulated by OPP in the brain

GenMAPP						
No.	MAPP name		Number changed	Percent changed	*Z* score	Permuted *P* value
1	Mm_Striated_muscle_contraction		3	7.1429	4.8690	0.0010
2	Mm_IL-6_NetPath_18		5	5.2083	5.1700	0.0030
3	Mm_Wnt_NetPath_8		4	3.7736	3.6930	0.0070
4	Mm_Delta-Notch_NetPath_3		3	3.8462	3.2330	0.0230
5	Mm_TGF-beta-Receptor_NetPath_7		4	2.8369	2.9570	0.0270
6	Mm_EGFR1_NetPath_4		4	2.4096	2.5620	0.0290
7	Mm_Insulin_Signaling		4	2.6316	2.7730	0.0320

Gene ontology						
**No.**	**GO name**	**GO type**	**Number changed**	**Percent changed**	***Z* score**	**Permuted** ***P*** **value**

1	Exocytosis	P	5	7.4627	6.9960	0.0000
2	Antimicrobial humoral response (sensu Vertebrata)	P	3	6.2500	4.8660	0.0010
3	Synaptic transmission	P	7	3.0172	4.5580	0.0020
4	Cell-cell signaling	P	9	1.8145	3.3180	0.0050
5	Nervous system development	P	10	1.7094	3.2900	0.0070
6	Potassium ion transport	P	4	2.7778	3.2190	0.0090
7	Synaptogenesis	P	2	9.0909	4.9590	0.0100
8	Neurotransmitter transport	P	2	4.8780	3.3960	0.0260
9	Cation transport	P	7	1.5317	2.4120	0.0270
10	Intracellular signaling cascade	P	13	1.1754	2.3000	0.0350
11	Protein amino acid dephosphorylation	P	3	2.4590	2.5180	0.0440
12	Transmembrane receptor protein tyrosine phosphatase activity	F	2	15.3846	6.6500	0.0020
13	Calcium ion binding	F	12	1.6173	3.4040	0.0030
14	Potassium channel activity	F	4	3.3613	3.7230	0.0040
15	Metal ion binding	F	29	0.9894	2.6160	0.0100
16	Protein tyrosine phosphatase activity	F	3	4.4118	3.8950	0.0110
17	Potassium ion binding	F	3	3.5294	3.3370	0.0170
18	Cation channel activity	F	5	2.1368	2.8800	0.0180
19	Protein binding	F	42	0.8891	2.5600	0.0180
20	Voltage-gated potassium channel activity	F	3	3.2967	3.1750	0.0210
21	Diacylglycerol binding	F	2	5.0000	3.4500	0.0320
22	Calmodulin binding	F	3	3.0612	3.0030	0.0320
23	GTP binding	F	5	1.6611	2.2310	0.0490
24	Synapse	C	6	4.1958	5.3380	0.0000
25	Cytoskeleton	C	11	1.4805	2.9280	0.0060
26	Membrane fraction	C	9	1.5306	2.7450	0.0110
27	Synaptosome	C	2	6.4516	4.0480	0.0130
28	Integral to plasma membrane	C	13	1.3458	2.8240	0.0130
29	Plasma membrane	C	18	1.1016	2.4560	0.0180

Gene ontology (GO) types: P, biological process; F, molecular function; C, cellular component.

**Table 2:** GenMAPPs and gene ontologies significantly down-regulated by OPP in the brain

GenMAPP						
No.	MAPP name		Number changed	Percent changed	Z score	Permuted *P* value
1	Mm_Prostaglandin_synthesis_regulation		3	9.6774	4.1950	0.0080
2	Mm_TNF-alpha-NF-kB_NetPath_9		6	3.5928	2.7330	0.0100
3	Mm_Alanine_and_aspartate_metabolism		2	15.3846	4.5510	0.0110
4	Mm_Integrin-mediated_cell_adhesion_KEGG		4	4.4444	2.7170	0.0190
5	Mm_T-Cell-Receptor_NetPath_11		5	4.0323	2.7860	0.0260
6	Mm_Focal_adhesion_KEGG		6	3.3149	2.5090	0.0260
7	Mm_Hedgehog_Netpath_10		2	9.5238	3.3850	0.0330
8	Mm_Valine_leucine_and_isoleucine_degradation		2	7.4074	2.8560	0.0390

Gene ontology						
**No.**	**GO name**	**GO type**	**Number changed**	**Percent changed**	***Z*** **score**	**Permuted** ***P*** **value**

1	Aldehyde metabolism	P	3	33.3333	10.3410	0.0000
2	Actin filament organization	P	3	6.6667	4.1200	0.0070
3	Hormone-mediated signaling	P	2	20.0000	6.4200	0.0080
4	Lipid metabolism	P	11	1.8933	2.6100	0.0110
5	Acute-phase response	P	2	8.6957	3.9770	0.0240
6	Neurotransmitter transport	P	2	4.8780	2.7130	0.0470
7	Intracellular signaling cascade	P	16	1.4467	2.0290	0.0480
8	Oxidoreductase activity	F	17	2.5641	4.6740	0.0000
9	Aldehyde reductase activity	F	2	40.0000	9.2900	0.0010
10	Rho GTPase activator activity	F	2	11.7647	4.7640	0.0060
11	Electron transporter activity	F	6	3.4286	3.5830	0.0060
12	Protein kinase binding	F	3	5.6604	3.6920	0.0130
13	GTPase activator activity	F	4	3.3613	2.8700	0.0210
14	Catalytic activity	F	54	1.1499	2.2540	0.0260
15	NADH dehydrogenase activity	F	2	6.4516	3.2900	0.0290
16	Isomerase activity	F	4	2.9412	2.5480	0.0310
17	NAD binding	F	2	6.6667	3.3620	0.0360
18	Lyase activity	F	4	2.6846	2.3330	0.0470
19	Membrane fraction	C	13	2.2109	3.4610	0.0010
20	Cytoplasm	C	48	1.3761	3.4600	0.0010
21	Endoplasmic reticulum	C	11	2.0794	2.9490	0.0050
22	Ruffle	C	2	8.6957	3.9770	0.0140
23	Focal adhesion	C	2	8.3333	3.8740	0.0200
24	Actin filament	C	2	8.0000	3.7770	0.0250
25	Cytosol	C	7	2.0290	2.2660	0.0270
26	Microsome	C	4	2.8986	2.5130	0.0380

Gene ontology (GO) types: P, biological process; F, molecular function; C, cellular component.

Most of the significant functions up-regulated by OPP in brains of mice are those involved in neurotrophic activity. For example, genes which are involved in calcium ion binding, calmodulin binding, potassium ion transport, and transmembrane receptor protein tyrosine phosphatase activity were up-regulated. Other genes involved in nervous system development, neurotransmitter transport, striated muscle contraction, synaptic transmission, and synaptogenesis were also up-regulated. These have implications for a role of OPP in increasing memory and reducing cognitive impairment.

**Figure 3:**
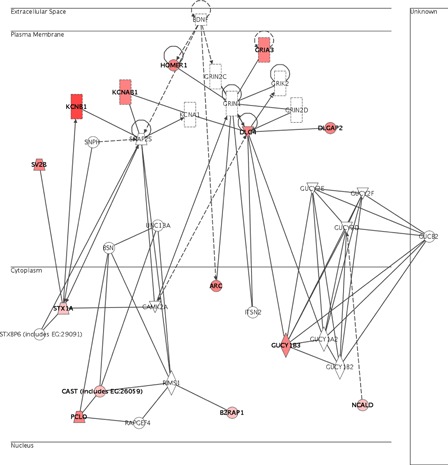
Genes up-regulated in the brain neurotrophic network. Genes identified as significantly up-regulated are colored red.

Interestingly, many of these genes were under the regulation of *Bdnf* (brain-derived neurotrophic factor), as shown in Fig. 3. For example, genes such as *Arc* (activity regulated cytoskeletal-associated protein), *Cast* or *D14Ertd171e* (DNA segment, Chr 14, ERATO Doi 171, expressed), *Gria3* (glutamate receptor, ionotropic, alpha-amino-3-hydroxy-5-methyl-4-isoxazolepropionic acid (alpha-amino-3-hydroxy-5-methyl-4-isoxazolepropionic acid) 3, alpha 3), *Kcnb1* (potassium voltage-gated channel, shab-related subfamily, member 1), *Kcnab1* (potassium voltage-gated channel, shaker-related subfamily, beta member 1), *Homer1* (homer homolog 1, *Drosophila*, transcript variant d), *Dlgap2* (discs, large homolog-associated protein 2, *Drosophila*), *Dlgh4* (discs, large homolog 4, *Drosophila*), *Sv2b* (synaptic vesicle glycoprotein 2 b), *Stx1a* (brain syntaxin 1A), *Gucy1b3* (guanylate cyclase 1, soluble, beta 3), *Ncald* (neurocalcin delta), *Bzrap1* (benzodiazapine receptor-associated protein 1), and *Pclo* (piccolo, presynaptic cytomatrix protein) are all under the regulation of *Bdnf*.

As inflammation has been implicated in brain aging, it was also interesting to find that genes involved in inflammation were down-regulated by OPP. In addition, genes involved in focal adhesion as well as alanine, aspartate, valine, leucine, and isoleucine metabolisms were down-regulated by OPP.

**Figure 4:**
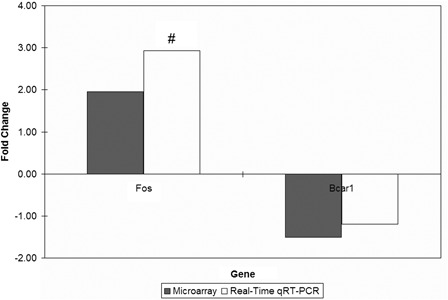
Gene expression fold changes of two target genes as determined by microarray and real-time qRT-PCR experiments. ^#^*P* < 0.05 for gene expression fold changes quantified by real-time qRT-PCR experiments as determined by two-tailed unpaired Student's *t*-test.

The fold changes obtained from the real-time qRT-PCR technique were comparable to those obtained from the microarray technique (Fig. 4), thus showing that the microarray data analysis carried out was reliable.

## Discussion

Many animal studies have shown that plant phenolics such as those from apple,^6^ berries,^2,4,7^ green tea,^8,9^ red wine,^10^ and pomegranate^11^ possess cognitive-enhancing effects. In addition, several plant extracts containing phenolic compounds such as those from *Polygala paniculata*^32^ and *Hypericum perforatum*^33^ were able to improve the rotarod performance of mice with induced Parkinson's disease. In the present study, the major components present in OPP are three isomers of caffeoylshikimic acid, caffeic acid, protocatechuic acid, and *p*-hydroxybenzoic acid. Although the compounds present in OPP were markedly different from these previously studied plant phenolics, the improved cognitive and motor functions found in mice given OPP in the present study suggest that OPP has similar neuroprotective properties. While the effects observed in the present study are mainly attributed to phenolic compounds, the possible effects of other components in OPP cannot be discounted, however. What is important here is that the extract in its entirety confers the outcomes reported in the present study.

In the long-term prevention and treatment of complex diseases, multi-component and multi-target botanical therapeutics administered through dietary interventions are particularly valuable.^34^ However, dietary interventions generally result in small effects on a big number of genes as compared to pharmaceutical interventions which elicit a larger but more targeted effect.^35^ As such, microarrays have become very important tools to dissect the molecular mechanisms by which antioxidants function and modulate gene expression,^36^ as they can fish out important regulated genes or biomarkers^37^ and detect a combined effect of several genes belonging to a similar biological pathway.^35^ In addition, phytochemicals such as phenolics are known to influence gene expression.^36^ Microarray analysis thus becomes an important tool to identify modulations of multiple gene networks caused by antioxidant micronutrients.^38^

The neurotrophic genes up-regulated by OPP are implied to have neuroprotective roles, and similar genes were found to be up-regulated by extracts of *Gingko biloba* leaves,^39^ which are already marketed as supplements to combat Alzheimer's disease, depression, short-term memory loss, as well as lack of attention and vigilance. Tyrosine phosphatases (*Ptprn* and *Ptprt*) are associated with the breakdown of intracellular neurofibrillary tangles, a hallmark lesion of Alzheimer's disease, while ionotropic glutamate receptor (*Gria3*) plays an integral role in synaptogenesis and the formation of neuronal circuitry.^39^ Both of these types of genes (*Ptprn*, *Ptprt*, and *Gria3*) were up-regulated by OPP.

The up-regulated *Bdnf* network represents a molecular mechanism by which OPP up-regulated genes involved in neuronal network maintenance and signaling. *Bdnf* influences the differentiation and survival of neurons and the maintenance of their arborizations.^40^ *Bdnf* is also essential to molecular mechanisms of synaptic plasticity, neural development, and brain cell survival.^41^ Although the *Bdnf* gene was not significantly up-regulated in the present study due to the filtering criteria used, it might play a probable role at the post-transcriptional or protein level. The BDNF protein would thus be an important target for future experiments investigating the neuroprotective effects of OPP.

The neuroplasticity associated gene, *Arc*, which is a direct transcriptional target of EGR (early growth response) transcription factors, was also up-regulated by OPP. Similar to the *Egr*s, the *Arc* gene is rapidly induced by synaptic activity, and its expression depends upon excitatory synaptic NMDA (*N*-methyl-d-aspartic acid) receptor activation and intracellular MAPK (mitogen-activated protein kinase) signaling. It has a critical role in maintaining long-term potentiation and long-term memory.^42^ *Dlgh4* or *Psd-95* (discs large homolog 4), is a post-synaptic marker which was significantly decreased by beta-amyloids but induced by withanosides from Ashwagandha (root of *Withania somnifera*), a herbal drug in ayurvedic medicine commonly used as a tonic and nootropic agent.^43^ Another gene up-regulated by OPP is *Fos* (Finkel–Biskis–Jinkins osteosarcoma), which is a marker of neuronal activity and plasticity. The age-related decline of *Fos* in the hippocampi of aged rats was also found to be attenuated by the ginsenoside Rg1 from *Panax ginseng*.^44^ The up-regulation of the *Arc*, *Dlgh4*, and *Fos* genes by OPP indicates that the extract enhances synaptic maturation, increases synaptic density, and induces local dendritically targeted protein synthesis in the brain, effects also known to be caused by increased *Bdnf* expression, as suggested by Yin *et al.*^40^

These possible cognitive-enhancing effects of OPP in the brain were also similar to the findings made by Lau *et al.*,^2^ in which blueberry antioxidants supplemented to Fisher 344 rats improved the cognitive performance of these animals as well as increased their brain neurogenesis and neuronal signaling. Hydroxytyrosol-rich olive mill wastewater extract was also found to protect murine-dissociated brain cells *in vitro* and *ex vivo* when they were challenged with ferrous ion-induced and nitric oxide-induced cytotoxicity, suggesting neuroprotective effects of the phenolic extract.^45^

As inflammation has been implicated in brain aging, it was also interesting to find that genes involved in inflammation, such as *Spp1* (secreted phosphoprotein 1 or osteopontin), *Saa3* (serum amyloid A3), and *Apod* (apolipoprotein D), were down-regulated by OPP supplementation in this study. *Spp1* is a pro-inflammatory protein reported to be up-regulated in several types of cancer and multiple sclerosis.^46^ The levels of serum amyloid A proteins which are members of the acute phase protein family, are increased in response to various injuries and have been implicated in the pathogenesis of chronic inflammatory diseases.^47^ On the other hand, *Apod* is associated with excitotoxic stress resulting in brain aging^48,49^ and Parkinson's disease.^50^ The down-regulation of these three genes in the brains of mice given OPP signifies that the extract possesses anti-inflammatory effects which can contribute towards the prevention of brain aging.

In addition, genes involved in focal adhesion were down-regulated by OPP. Focal adhesion is a mechanism embodying the actin and cytoskeleton cell connections of a cell to the extracellular matrix. One of these genes, *Actb* (beta-actin), encoding a cytoskeletal protein found to be elevated in the brains of Alzheimer's disease patients and in reactive glia, was noted to be down-regulated. This was also seen by Deshane *et al.*^51^ in the brains of mice fed a grape seed extract enriched with proanthocyanidins.

Genes involved in alanine, aspartate, valine, leucine, and isoleucine metabolisms were also down-regulated by OPP. Ramassamy^52^ has suggested that the prevention of the oxidation of some of these amino acids by *Gingko biloba* may have helped prevent the formation of beta-amyloid fibrils in Alzheimer's disease. Other functions down-regulated by OPP include electron transporter activity, guanosine triphosphatase activator activity, and intracellular signaling cascade. *Dnajc4* (*DnaJ Hsp40* homolog, subfamily C, member 4) which encodes a chaperone protein, was also down-regulated by OPP. In experiments conducted by Lee *et al.*,^48^ a related gene *Hsp40* (*DnaJ* homolog 1) which was up-regulated by aging in the brains of mice, was attenuated 100% by caloric restriction. In addition, two homeobox genes (*Hoxa5* and *Hoxb5*) were turned off in the present study as their expression in the brain could not be detected after the supplementation of OPP. *Hoxb5* was found up-regulated in incipient Alzheimer's disease.^53^

The up-regulation of genes involved in brain development and activity as well as the down-regulation of genes involved in inflammation in the brains of mice thus imply that OPP may have some neuroprotective and anti-inflammatory effects. These microarray results are in agreement with the results of epicatechin supplementation to C57BL/6 mice, in which genes involved in learning were up-regulated while those involved in inflammation were down-regulated.^9^

We acknowledge that the biggest limitation in this study is the fact that brain histology coupled with protein measurements had not been carried out. However, this present study was meant to be exploratory rather than confirmatory as no studies on the effects of OPP on the brain were reported before this. After carrying out the water maze and rotarod trials, we found that OPP had effects on the brain which could manifest physiologically. As whole genome microarrays would be able to detect the expression changes of a large number of genes, we then decided to carry out transcriptomic analysis on the brains and identify the gene expression changes which might provide initial clues to help explain how OPP confers these neuroprotective effects. We hope that based on the present study, we could further extend the findings by carrying out experiments on other animal models in the future, such as mice genetically modified to have brain-related diseases. In these models, immunohistochemistry carried out on the brain to detect BDNF would be more meaningful to confirm the proteins and brain structures affected by OPP.

In conclusion, we found that OPP has significant neuroprotective properties as it improved the cognitive and motor functions of mice on a normal diet. These improvements can be attributed to the up-regulation of synaptogenesis and neurotransmission genes as well as the down-regulation of inflammatory genes caused by the compounds. This study also implies the possible application of OPP in the prevention and treatment of neurodegenerative diseases such as Alzheimer's and Parkinson's.
